# Association of Pulmonary Manifestations in Leptospirosis: A Cross-Sectional Study

**DOI:** 10.7759/cureus.87656

**Published:** 2025-07-10

**Authors:** Kadavanoor Srijith, Koothupalakkal Ravi Rajesh, Thuruthiath Nisha

**Affiliations:** 1 Gastroenterology, Government Medical College Hospital, Thrissur, IND; 2 Infectious Diseases, Government Medical College Hospital, Thrissur, IND; 3 Otolaryngology, Amala Institute of Medical Sciences, Thrissur, IND

**Keywords:** acute renal failure, acute respiratory distress syndrome, leptospirosis, pulmonary hemorrhage, zoonotic disease

## Abstract

Background: The symptoms of leptospirosis can range from mild pulmonary symptoms to acute respiratory distress syndrome (ARDS) and lung hemorrhage. As a result, a delayed diagnosis may have negative consequences. A cross-sectional study was conducted to determine the prevalence and spectrum of pulmonary manifestations in leptospirosis.

Methods: This cross-sectional study was conducted among patients admitted with febrile illness and diagnosed with leptospirosis by IgM anti-leptospiral antibodies. Clinical features, with special focus on pulmonary complaints, were recorded using a questionnaire. Presentations such as jaundice, oliguria, cough, hemoptysis, breathlessness, chest pain, evidence of ARDS, and pulmonary hemorrhage were noted. Chest X-rays were assessed for the presence of reticular infiltration and airspace nodules. The data were subjected to statistical analysis.

Results: The study included 50 patients (aged 18 to 80 years) who had been diagnosed with leptospirosis. Among the 50 patients, 42 (84%) were male; the mean age of the patients was 43.7 years. All patients experienced fever, followed by myalgia, which was noted in 45 (90%) patients. In 22 (44%) patients, pulmonary symptoms - including cough, breathlessness, and hemoptysis - were identified. Abnormal chest X-rays were noted in 10 (20%) patients, and the most common pattern that increased the likelihood of requiring artificial breathing was airspace nodules. Tachypnea and hemodynamic impairment were two parameters that significantly correlated with abnormal chest X-rays. There were 16 deaths, with the leading cause of death being pulmonary problems in 13 patients (81.25%), followed by acute renal failure in two patients (12.5%) and multi-organ dysfunction in one patient (6.25%).

Conclusion: Leptospirosis frequently manifests as pulmonary symptoms, which are linked to a high death rate. Reducing mortality requires early detection and treatment.

## Introduction

Leptospirosis, which often occurs as large outbreaks around the world, is caused by *Leptospira interrogans*. Weil, in 1886, described capillary vasculitis as the cause of hepatic, renal, and pulmonary involvement in leptospirosis. Humans typically contract leptospirosis, an emerging zoonotic disease, by coming into contact with soil or water tainted with rat urine. It generally manifests as flu-like symptoms accompanied by modest renal and hepatic impairment. The disease is characterized by a biphasic pattern, with an immunological phase that lasts one week following an acute/septicemic phase that also lasts one week. Antibody synthesis and leptospire excretion in urine are indicators of the immunological phase. Only a small percentage of patients experience a fulminant monophasic disease as the predominant clinical course, rather than the typical biphasic sickness. These patients begin with an acute, nonspecific disease that quickly develops into extensive lung hemorrhage, refractory shock, jaundice, and renal failure [1]. Laboratory, clinical, and epidemiological characteristics are used to make the diagnosis. The diagnosis is frequently overlooked in patients who have atypical symptoms [2]. The serological tests have been classified as serovar-specific tests and genus-specific tests [3]. Typically, pulmonary involvement occurs during the immunological phase, and 20-70% of patients experience overt pulmonary symptoms, most of which resolve without any long-term effects [4].

The main pulmonary symptoms that patients with leptospirosis may exhibit include cough, chest pain, dyspnea, mild to severe hemoptysis, and acute respiratory distress syndrome (ARDS). The fourth to sixth day of illness is when the pulmonary symptoms typically manifest. The condition can progress very quickly and can be fatal within 72 hours [5]. Due to their general nature and overlap with other tropical diseases, these signs are often underdiagnosed, leading to delays in receiving the proper therapy. Hemoptysis, a manifestation of pulmonary hemorrhage, has been reported to be present in 17-50% of patients [6]. In a study by Martínez et al., no correlation was found between oliguria and pulmonary rales, suggesting that lung involvement may not be secondary to hypervolemia caused by oliguric acute renal failure [7]. Due to inadequate monitoring systems and diagnostic capacities in environments with low resources, the actual incidence of pulmonary problems is likely underreported. This study aimed to determine the prevalence and spectrum of pulmonary manifestations in leptospirosis.

## Materials and methods

Study design and population

A cross-sectional study was conducted among patients (over the age of 18 years) with febrile illness diagnosed with leptospirosis, admitted to the Department of Medicine, Government Medical College Hospital, Central Kerala, South India. The study was carried out over a period of six months, from June 2011 to November 2011. All patients presenting with fever, myalgia, and headache, associated with at least one of the following symptoms and signs - jaundice, oliguria, cough, hemoptysis, breathlessness, meningeal signs with altered sensorium, and bleeding tendencies including conjunctival suffusion - were screened for IgM leptospiral antibodies and included in the study. Patients with chronic renal or hepatic disease, chronic debilitating illness, or other causes of febrile illness were excluded. Written informed consent was obtained from all participants. The study was approved by the Institutional Ethics Committee (reference no. IEC/GMCTSR/43/2011).

Study procedure

Patients who were clinically diagnosed with leptospirosis and subsequently confirmed by IgM enzyme-linked immunosorbent assay (ELISA) were evaluated through medical history, physical examination findings, and laboratory investigations focusing on pulmonary problems. Laboratory data included complete blood count with erythrocyte sedimentation rate (ESR), peripheral smear, random blood sugar (RBS), renal function tests, liver function tests, serum electrolytes, urine routine analysis, and arterial blood gas (ABG) analysis. Pulmonary manifestations were defined as the presence of cough, breathlessness, chest pain, hemoptysis, evidence of ARDS, pulmonary hemorrhage, presence of infiltrates on chest X-ray, and patients requiring mechanical ventilation. ARDS was defined as a clinical syndrome characterized by severe dyspnea of rapid onset, hypoxemia evidenced by partial pressure of arterial oxygen (PaO_2_)/fraction of inspired oxygen (FiO_2_) ≤300 mmHg (with PaO_2_ measured in mmHg and FiO_2_ between 0.21 and 1.00), and the presence of diffuse pulmonary infiltrates leading to respiratory failure [8]. Pulmonary hemorrhage was defined as the presence of hemoptysis >300 mL or aspiration of fresh blood after endotracheal intubation that did not clear with suctioning, accompanied by respiratory insufficiency [8].

The chest X-ray was assessed for the presence of reticular infiltration, airspace nodules, consolidation, cardiomegaly, evidence of congestive heart failure, cavity formation, atelectasis, hilar adenopathy, and pleural effusions. The data were collected and analyzed.

Statistical analysis

Statistical analysis was performed using IBM SPSS Statistics v16 (SPSS Inc., Chicago, USA). Categorical values were expressed as frequency with percentages in parentheses. Fisher's exact test was used to compare observations, with p<0.05 considered significant.

## Results

A total of 50 patients were studied, comprising 42 male (84%) and eight female (16%) patients. The age of the study population varied from 21 to 90 years; the majority of the patients were aged between 41 and 60 years (56%), with a mean age of 43.7 years.

Fever was observed as the most common manifestation in almost all patients. Myalgia was seen in 45 (90%) patients. Jaundice was identified in 22 patients (44%), and oliguria was present in 21 patients (42%). Respiratory symptoms included cough in 16 patients (32%), breathlessness in 15 patients (30%), and hemoptysis in six patients (12%). None of the patients reported chest pain. Other less common manifestations included diarrhea in seven (14%) patients, vomiting in five (10%) patients, and altered sensorium in two (4%) patients. Overall, 22 patients (44%) exhibited respiratory symptoms (see Table 1).

**Table 1:** Frequency distribution of clinical manifestations of leptospirosis

Variable	Frequency
Vomiting	5 (10%)
Altered sensorium	2 (4%)
Diarrhea	7 (14%)
Hemoptysis	6 (12%)
Chest pain	0
Breathlessness	15 (30%)
Cough	16 (32%)
Oliguria	21 (42%)
Jaundice	22 (44%)
Myalgia	45 (90%)
Fever	50 (100%)
Respiratory symptoms	22 (44%)

During the general examination, conjunctival congestion was found in 45 (90%) patients, and icterus was noted in 22 (44%) patients. Subconjunctival hemorrhage was seen in eight (16%) patients, and a single patient presented with a generalized rash. A total of 17 (34%) patients had tachycardia (pulse rate >100 bpm). Fourteen (28%) patients exhibited hypotension (systolic blood pressure <90 mmHg) at the time of presentation, while 15 (30%) patients had severe tachypnea (respiratory rate >30 breaths per minute), and 12 (24%) patients had oxygen saturation levels below 90%. Chest auscultation revealed that nine (18%) patients had crepitations (see Table 2).

**Table 2:** General examination findings SpO_2_: Oxygen saturation; RR: Respiratory rate; SBP: Systolic blood pressure; PR: Pulse rate

Variable	Frequency
Conjunctival congestion	45 (90%)
Subconjunctival hemorrhage	8 (16%)
Icterus	22 (44%)
Rash	1 (2%)
SpO_2_ <90%	12 (24%)
RR >30 breaths per minute	15 (30%)
SBP <90 mmHg	14 (28%)
PR >100 bpm	17 (34%)
Crepitations on chest auscultation	9 (18%)

A total leucocyte count of more than 15,000/cm^3^ was observed in 16 patients (32%). Thrombocytopenia (platelet count <100,000/cm^3^) was noted in 41 patients (82%). Four patients (8%) displayed hyperglycemia (random blood sugar >200 mg/dL). Elevated serum creatinine levels (>2 mg/dL) were found in 40 patients, and hypokalemia was observed in five patients (10%). Raised serum bilirubin levels (>10 mg/dL) were seen in 11 patients (22%), and 23 patients (46%) had abnormal ABG results. Nineteen patients had metabolic acidosis, while two patients had respiratory acidosis and two patients had combined metabolic and respiratory acidosis (see Table 3).

**Table 3:** Laboratory investigation data RBS: Random blood sugar; ABG: Arterial blood gas

Variable	Frequency
Total bilirubin >10 mg/dL	11 (22%)
Serum potassium <3.5 mEq/L	5 (10%)
Serum creatinine >2 mg/dL	40 (80%)
RBS >200 mg/dL	4 (8%)
Platelet count <1 lakh/cm^3^	41 (82%)
Abnormal ABG	23 (46%)

Abnormal chest X-ray findings were noted in 10 patients (20%), among whom eight had airspace nodules, one patient had reticular infiltrates, and one patient exhibited features of cardiac failure. Of the 50 patients, 12 (24%) had evidence of ARDS, 13 patients (26%) were on mechanical ventilation, and five patients (10%) exhibited evidence of pulmonary hemorrhage. Mortality was observed in 16 cases (32%) (see Table 4).

**Table 4:** Distribution of respiratory manifestations ARDS: Acute respiratory distress syndrome

Variable	Frequency
Pulmonary hemorrhage	5 (10%)
Mechanical ventilation	13 (26%)
ARDS	12 (24%)
Deaths	16 (32%)

A comparison of the clinical findings is presented in Tables 5-6. Tachypnea with a respiratory rate >30 breaths per minute was identified as a significant predictor (Fisher's exact test <0.05) of mortality. The presence of crepitations was also a significant predictor (Fisher's exact test <0.05) of mortality. Oliguria showed no relation to the presence of crepitations on chest auscultation (Fisher's exact test >0.05). There was a significant relationship between the presence of respiratory symptoms and ARDS (Fisher's exact test <0.05). The presence of airspace nodules indicated severe leptospirosis and was significantly associated with the need for mechanical ventilation (Fisher's exact test <0.05). Patients presenting with respiratory symptoms were more likely to have an abnormal chest X-ray (Fisher's exact test <0.05). Patients with hemodynamic compromise at presentation had a higher likelihood of having an abnormal chest X-ray (Fisher's exact test <0.05). Additionally, patients with tachypnea (respiratory rate >30 breaths per minute) at presentation had a greater chance of having an abnormal chest X-ray (Fisher's exact test <0.05). Patients with desaturation at presentation were also more likely to have an abnormal chest X-ray (Fisher's exact test <0.05). Elevated serum creatinine levels (>2 mg/dL) did not show a relationship with the likelihood of having an abnormal chest X-ray (Fisher's exact test >0.05).

**Table 5:** Comparison of clinical manifestations Fisher's exact (p-value) <0.05 was considered statistically significant. * indicates p-value <0.05. ARDS: Acute respiratory distress syndrome

Manifestation	Findings			P-value (Fisher’s Exact Test)
Respiratory rate >30 breaths per minute	Recovered	Expired	Total	
Absent	33	2	35	<0.0001^*^
Present	1	14	15	
Total	34	16	50	
Crepitations	Recovered	Expired	Total	
Absent	34	7	41	0.0000045661^*^
Present	0	9	9	
Total	34	16	50	
Oliguria	Crepitations absent	Crepitations present	Total	
Absent	26	3	29	0.100456
Present	15	6	21	
Total	41	9	50	
Respiratory symptoms	No evidence of ARDS	Evidence of ARDS	Total	
Absent	27	1	28	0.0000866781^*^
Present	11	11	22	
Total	38	12	50	
Airspace nodules	No mechanical ventilation needed	Mechanical ventilation needed	Total	
Absent	37	5	42	0.0000023972^*^
Present	0	8	8	
Total	37	13	50	

**Table 6:** Comparison of clinical manifestations Fisher's exact (p-value) <0.05 was considered statistically significant. * indicates p-value <0.05. SBP: Systolic blood pressure; SpO_2_: Oxygen saturation

Manifestation	Findings			P-value (Fisher’s Exact Test)
Respiratory symptoms	Normal chest X-ray	Abnormal chest X-ray	Total	
Absent	28	0	28	0.0000629506^*^
Present	12	10	22	
Total	40	10	50	
SBP >90 mmHg	Normal chest X-ray	Abnormal chest X-ray	Total	
Absent	7	7	14	0.0025767841^*^
Present	33	3	36	
Total	40	10	50	
Respiratory rate >30 breaths per minute	Normal chest X-ray	Abnormal chest X-ray	Total	
Absent	34	1	35	0.0000173455^*^
Present	6	9	15	
Total	40	10	50	
SpO_2 _<90%	Normal chest X-ray	Abnormal chest X-ray	Total	
Absent	37	1	38	0.0000008203^*^
Present	3	9	12	
Total	40	10	50	
Serum creatinine	Normal chest X-ray	Abnormal chest X-ray	Total	
Less than 2 mg/dL	10	0	10	0.0825192342
More than 2 mg/dL	30	10	40	
Total	40	10	50	

## Discussion

Results of the study found that all patients experienced fever, which was followed by myalgia noted in 45 (90%) patients. In 22 (44%) patients, pulmonary symptoms were identified. Ten patients (20%) had abnormal chest X-rays, with airspace nodules being the most prevalent pattern. These patients were more likely to need mechanical ventilation (Fisher's exact test <0.05). Tachypnea and hemodynamic impairment were parameters that significantly correlated with abnormal chest X-rays (Fisher's exact test <0.05). There were 16 deaths, and the leading cause of death was pulmonary complications, seen in 13 (81.25%) patients, followed by acute renal failure in two (12.5%) patients, while one patient died due to multi-organ dysfunction.

Progression of the disease and development of its complications are seen in cases where there is a delay in diagnosis [5]. The most common clinical presentation of leptospirosis includes fever, jaundice, and renal failure [5]. Around 20-70% of patients may experience pulmonary symptoms that are usually mild and resolve without any sequelae [9]. Philip et al. in their study on the clinical manifestations developed in hamsters infected with the *Leptospira* ​​​​*interrogans* strain HP358 described that sudden overexpression of pro-inflammatory cytokines led to severe leptospirosis. Rapid damage in the lungs, liver, and kidneys was preceded by the early occurrence of hemorrhage in the lungs [10].

The pulmonary manifestations may range from mild respiratory symptoms to severe conditions such as ARDS and pulmonary hemorrhage. The most common pulmonary symptoms include cough (either dry or productive), hemoptysis, and dyspnea. This study primarily intended to determine the prevalence, characteristics, and severity of pulmonary involvement in leptospirosis. In our study, cough, breathlessness, and hemoptysis were the common respiratory symptoms. In a study by Paganin et al., out of a total of 169 patients with leptospirosis, 134 (79.2%) had respiratory symptoms, with cough noted in 71 (53.3%), dyspnea in 53 (40.1%), and hemoptysis in 58 (45.3%) patients, respectively [11].

The higher incidence of pulmonary symptoms may be due to differences in the infecting serovars, which may explain the lower incidence of jaundice in our study. Tachypnea and the presence of crepitations on chest auscultation were found to be significant predictors of mortality (Fisher's exact test <0.05). In our study, there was no association between oliguria and pulmonary manifestations (Fisher's exact test >0.05), suggesting that lung involvement is unrelated to hypervolemia secondary to acute renal failure. An elevated serum creatinine of >2 mg/dL did not have an association with abnormal chest X-ray (Fisher's exact test >0.05). This suggests that respiratory involvement is indeed a separate entity and not a part of renal dysfunction. The observation that pulmonary presentations can occur independently without overt renal and liver impairment implies that severe pulmonary forms of leptospirosis may be considered a form of leptospirosis separate from Weil’s disease [12].

The presence of airspace nodules and reticular patterns in chest X-rays was the most frequently observed abnormal radiographic finding in patients with respiratory symptoms in our study. Airspace nodules were also the most common abnormality noted in a study done by Im et al. [13]. It was noted in our study that airspace nodules detected on chest radiographs had a significant association with the need for mechanical ventilation (Fisher's exact test <0.05). Few other studies have shown that the presence of alveolar infiltrates is associated with mortality [14,15]. The presence of respiratory symptoms, hemodynamic compromise (systolic blood pressure <90 mmHg), tachypnea, and peripheral oxygen saturation (SpO_2_) <90% at presentation were all significantly associated with abnormal chest X-ray findings in our study (Fisher's exact test <0.05). Oliguria, hypotension, and abnormal chest auscultation were found to be independent predictors of severe leptospirosis mortality [15,16]. In our study, out of the 50 leptospirosis patients, there were 16 deaths with a case fatality rate of 32%. Although the case fatality rate was high, it may be attributed to pulmonary complications. In a study, 74% of 89 fatal cases of leptospirosis had clinically detectable pulmonary involvement, which is the strongest predictor of death [17].

During an epidemic season, individuals with respiratory symptoms should be closely monitored for leptospirosis. Given their high mortality rate, they should receive treatment as soon as possible and be referred to a better facility. The initial examination of these patients should involve a chest radiograph. Chest radiography may reveal airspace nodules, which could be a sign of severe leptospirosis.

Limitations

The total number of patients studied was less. Some cases with a clinical suspicion of pulmonary leptospirosis that appeared early in the course of illness, when IgM ELISA may be negative, could not be included because IgM ELISA was used to confirm leptospirosis. The majority of these individuals had negative IgM ELISA results, and they all succumbed within 24 hours of admission. Therefore, it was impossible to determine the true prevalence of pulmonary leptospirosis. Since the majority of cases we examined in the tertiary care facility came from primary care facilities, it was not possible to study fewer manifestations. Most cases examined either had pulmonary symptoms or hepatorenal syndrome; thus, situations like anicteric leptospirosis may have gone unnoticed. Additionally, since the microscopic agglutination test could not be performed, identifying the infecting serovars was not possible.

## Conclusions

Pulmonary manifestations such as ARDS and pulmonary hemorrhage are common in leptospirosis. The most frequently observed respiratory symptom is cough, followed by breathlessness and hemoptysis. Tachypnea and the presence of lung crepitations are significant predictors of mortality. The presence of airspace nodules indicates severe leptospirosis in patients requiring mechanical ventilation. Factors associated with an increased likelihood of an abnormal chest X-ray include the presence of respiratory symptoms, tachypnea, hemodynamic compromise, and SpO_2_ of <90% at presentation.
